# Interprofessional collaborative practice in health and social care for people living with multimorbidity: a scoping review protocol

**DOI:** 10.1186/s13643-024-02730-x

**Published:** 2025-01-02

**Authors:** Josephine-L. K. Murray, Virginia Hernandez-Santiago, Frank Sullivan, Joanna Hornal, Farhana Badshah, Ben Keatley, Jillian Galbraith, Pam Channer, Anne Fearfull, Anne Haddow, Eleanor Johnston, Maureen Ward, Veronica O’Carroll

**Affiliations:** School of Medicine, University of St Andrews, North Haugh, St Andrews, KY16 9TF UK

**Keywords:** Scoping review, Multimorbidity, Interprofessional collaborative practice, Healthcare research, Healthcare public health, Social care research, Health and social care workforce

## Abstract

**Background:**

Multimorbidity, the co-existence of two or more conditions within an individual at any one time, is globally increasing and forecasted to rise. This poses a significant challenge for current models of healthcare delivery, which are now ill-equipped to meet the future population health needs. Interprofessional collaborative practice is a specific way professionals work closely together and with patients and their families to improve patient outcomes. Evidence suggests it can improve outcomes for people living with a single condition. What remains unknown is if interprofessional collaborative practice has been used to improve the outcomes of people living with multimorbidity, and if so, to what extent?

**Methods:**

A scoping review is proposed to identify prior peer-reviewed research and grey literature related to interprofessional collaborative practice for multimorbidity in health and social care settings.

A search strategy will identify primary, peer-reviewed research and grey literature. An initial limited search will be conducted to identify relevant existing systematic reviews. Their methods will be examined and their search terms scrutinised. A second comprehensive search will be used to interrogate four databases, looking back 10 years, seeking articles published in English, French, Spanish or Portuguese. Hand searching will be performed on all included full-text articles for any articles missing from the two steps above.

Critical data will be extracted by adapting existing data abstraction forms based on the needs of the research objectives. These forms will be piloted before use.

The results will be analysed descriptively. If appropriate, qualitative content analysis may be undertaken. Where sufficient numbers of homogeneous interventions exist, meta-analysis techniques will be applied.

Results will be presented in tabular, graphic, and diagrammatic information displays.

**Discussion:**

This scoping review will provide an overview of the current evidence base of interprofessional collaborative practice used internationally for people living with multimorbidity in health and social care settings. These findings will provide valuable information to improve health and social care practice as well as change systems and policy to meet the population need of multimorbidity.

**Systematic review registration:** The protocol was submitted to Open Science Framework on 19 December 2023 and registered on OSF Registries. Registration DOI: 10.17605/OSF.IO/UXHG3.

**Supplementary Information:**

The online version contains supplementary material available at 10.1186/s13643-024-02730-x.

## Background

Multimorbidity (MM) is a significant cause of decreased life expectancy, loss of quality of life, increased need for social and informal care and burden on healthcare systems worldwide [1]. MM was first quantified as affecting 23% of the Scottish population; however, more recent estimates indicate that by the year 2035, prevalence in the UK is expected to double, with an increase mainly in people who live with four or more conditions [2, 3]. Internationally, this has been cited as the greatest public health problem facing the world today [4].

The burden MM places on both healthcare services and people living with MM is significant [5]; disorganisation and fragmentation of healthcare are the primary barriers to delivering patient-centred care and shared decision-making [6].

Interprofessional collaborative practice (IPCP) occurs in health and social care when individuals from different professional backgrounds work interdependently and in an integrated way to provide comprehensive services by working with patients, their families, carers and communities to deliver the highest quality of care across settings [7].

Healthcare interventions which improve outcomes for those with MM have cited IPCP as an integral component [8]. Yet, to date, any research in this area has only considered health and social care provision changes from professionals’ perspectives. What remains unknown is what IPCP interventions have been tried to improve health and health services for patients of the multimorbid population and to what extent IPCP effectively improves health and health service outcomes for this multimorbid population.

### What is multimorbidity?

While challenging to define, multimorbidity (MM) is broadly defined as ‘the existence of two or more health conditions in an individual, atany onetime’ [9]. This definition is generally accepted — and has been validated through an umbrella review regarding how to define multimorbidity. However, as a working definition, this is problematic, as it would include all comorbidities and multimorbidity.

While the literature and definitions of people living with comorbidities started in the 1970s [10], Van den Akker first made the differentiation between multimorbidity and comorbidity in a review paper in1996 [11].

There appears to be a paucity of primary research on this topic. However, the existing peer-reviewed literature suggests the key differences are that in comorbidities there is an index condition (for example diabetes as an index condition and diabetic retinopathy as a comorbidity); if the index condition is treated, the other comorbidities will also improve, and if the index condition is measured, this can guide the treatment and management of other concurrent conditions [12].

Furthermore, contrastingly, multimorbidity has no index condition; each concurrent long-term condition has no priority over another co-existing disease. Additionally, the patient, rather than the disease, is the focus of treatment, measurement and management [13, 14].

### What is interprofessional collaborative practice?

IPCP is internationally defined as ‘whenmultiple health workers from different professional backgrounds provided comprehensive services by working with patients, their families, carers and the communities to deliver the highest quality of care across settings’ [7]. Collaborative practice is a specific form of teamwork that emerges from and relies on the knowledge and skills of two or more team members, including healthcare professionals, social care professionals, public health professionals, communities, patients and families [15]. IPCP can be defined as the above, specifically where individuals from different professional groups form a partnership as a team of health providers with a patient in a participatory, coordinated, integrated and interdependent manner to share goals, accountability, decision-making around health and social care issues, therefore enhancing high-quality care and improve patient outcomes [7–19].

An integrative literature review comparing the differences between multidisciplinary, interdisciplinary and interprofessional practice concluded that ‘multi’ refers to teams of different disciplines who may work consecutively, treating clients. Still, they share only information, whereas ‘inter’ has more formal structures and share decision-making, conflict resolution and interdependence. This integrative review also concluded that multidisciplinary was the most frequently used terminology in healthcare team peer-reviewed research [20].

The differences between ‘interprofessional’ and ‘multidisciplinary’ have been helpfully highlighted and stratified in attitudes, knowledge and behaviours by Steffin and colleagues [21]. These authors further argue that multidisciplinary team members are focused solely on the patient’s outcome. They further claim that multidisciplinary teams are not concerned with how effectively the team functions, and that multidisciplinary teams are hierarchically organised, and the individuals from the discipline with the most agency are commonly identified as a team leader. They claim that interprofessional teams contrast in that they have shared leadership, and that discipline-specific roles and responsibilities need not apply. They point out that interprofessional team members have an in-depth knowledge of each other’s training and skill sets. These pave the foundation for advanced team skills of role mapping, group processing, advanced communication and superior conflict navigation. It is for these reasons that the authors claim that IPCP is the gold standard for the care of complex patients with chronic diseases [21].

### Outcomes of interprofessional collaborative practice

A Cochrane review compared the effectiveness of IPCP with standard care and also IPCP with other interventions. They examined four different IPCP interventions:

1. Externally facilitative interprofessional activities
2. Interprofessional rounds
3. Interprofessional meetings
4. Interprofessional checklists

They considered three key outcomes: patient health outcomes, clinical process outcomes and collaborative behaviour. They report that having reviewed nine randomised trials, there may be improvements in the efficiency of care, clinical processes and patient outcomes. Unfortunately, the review also highlights that due to the risk of bias in the studies, the quality of evidence was low, and more robust research is required in this field [22].

While the Cochrane review did not consider morbidity, mortality or patient satisfaction, a systematic review by Roskam highlighted that collaboration between professional groups has improved these outcomes, increased job satisfaction for the workforce and decreased health costs. The review also highlighted several barriers to realising the benefits of IPCP in practice. These barriers included historically entrenched views; perceptions of professional dominance; clinical uncertainty; poor communication; limited time and resources, as well as a lack of institutional support; and a resistance to change [22, 23].

### Health and social care

The World Health Organization recognises that achieving its ambitions of universal health coverage and Sustainable Development Goals depends on the availability, accessibility, acceptability and quality of its health workforce. However, it highlights a projected shortfall in the workforce of 10 million worldwide by 2023. Further contributing to the shortfall includes the mismatch between education and employment for the healthcare system and population needs [24].

Social care refers to a range of services and support designed to help individuals who need extra assistance in their daily lives. This can include personal care, practical help and emotional support. It has been defined as follows:

‘Any of numerous publicly or privately provided services intended to aid disadvantaged, distressed, or vulnerable persons or groups’ [25].

Literature suggests that social care needs play a vital role for people living with multimorbity [26]. However, it is recognised that the majority of evidence to date has focused solely on health data, and therefore has not necessarily been able to meet the needs or improve the outcomes of people living with multiple long-term conditions. Failure to include social care may be a contributing reason for the lack of success in interventions for improvement for those living with multimorbidity [27]. Therefore, this review intends to include both health and social care sectors.

### Justification

This scoping review is being carried out to support a research study in Scotland.

Healthcare provision in the National Health Service (NHS) in Scotland has always been free at the point of delivery; however, due to general life expectancy increases, associated increased levels of morbidity, the burden of disease and the cost of healthcare continuing to rise, the future of the current healthcare system is sometimes considered as unsustainable [28]. This matter is further exacerbated, as the NHS was initially designed to cater for ‘single diseases’ and mainly for acute presentations, yet the population now presents to the healthcare system with *multimorbidity*— requiring care for the coexistence of two or more long-term conditions [29].

The most significant financial cost to the healthcare system in Scotland is human resources [30]. Recent reports state that the social care workforce is undervalued, badly paid for vital, skilled work and held in low esteem in comparison particularly to the health workforce [31]. Therefore, evidence-based ways of enhancing the effectiveness of the NHS and Social care workforce in all care settings should be considered to provide sustainable care models for the future [32].

## Methods/design

### Study aim

This scoping review aims to examine what is already known about IPCP for multimorbidity and identify gaps in the current relevant literature.

### Why a scoping review?

It has been established that researchers who wish to explore the extent of existing literature and identify gaps in current research activity should carry out a scoping review [33–35]. It is essential to ensure this work has not already been carried out.

A preliminary search of MEDLINE, the Cochrane Database of Systematic Reviews, *JBI Evidence Synthesis* and Google Scholar was conducted, and no current or planned systematic reviews or scoping reviews on the topic were identified.

The identified reviews examined comorbidities or multidisciplinary working, but none reviewed IPCP specifically for multimorbidity. Any that considered either multimorbidity or IPCP only considered health settings rather than health and social care settings.

The objective of this scoping review is to assess the extent of the literature on the use and success of IPCP within health and social care for people living with multimorbidity.

### Review question

How has IPCP been used in health and social care settings to make improvements for people living with multimorbidity?

#### Subquestions

- What are the demographics and diagnostic groupings in which IPCP has been used for multimorbidity?
- What are the professional group combinations which have been working interprofessionally for multimorbidity?
- In what geographical, policy and clinical/social contexts has IPCP been used for multimorbidity?
- What scientific methods have been used to measure the success of IPCP for multimorbidity?

### Eligibility criteria

#### Participants

The participants for this scoping review are those living with multimorbidity, defined as ‘the existence of two or more long-term health conditions in an individual, at any one time’, in all ages, sexes and ethnicities. Participants would be excluded if they had comorbidities, where there is an index condition, as described elsewhere in the article (please see the ‘Glossary’).

#### Concept

The concept for this scoping review is interprofessional collaborative practice defined as follows:

‘When multiple health workers from different professional backgrounds provide comprehensive services by working with patients, their families, carers and communities to deliver the highest quality of care across settings’ [7].

Any professional belonging to the following regulated professional groups is within the conceptual definition (although this list is for UK professionals only, in other countries, other professional groups may also exist): art therapist, chiropodist/podiatrist, dentist, dietitian, drama therapist, medical doctor, midwives, music therapist, nursing, occupational therapist, operating department practitioner, orthoptist, osteopath, paramedic, pharmacist, play therapist, physiotherapist, psychologists, prosthetist/orthotist, radiographer, social worker and speech and language therapist.

This review excludes nonregulated professional groups and professionals working in a multidisciplinary way rather than an interprofessional collaborative way. They must also work with their patient and their caregiver/family.

#### Context

The context for this scoping review is health and/or social care. This encompasses all geographies, policy landscapes, all health systems, all social care systems, all management structures, all logistical and financial models and all clinical settings. This is deliberately designed to be as inclusive as possible.

#### Types of sources

This scoping review will consider experimental and quasi-experimental study designs, including randomised controlled trials, non-randomised controlled trials, before-and-after studies and interrupted time-series studies. In addition, the following studies and papers will also be considered for inclusion:

- Analytical observational studies, including prospective and retrospective cohort studies, case–control studies and analytical cross-sectional studies, will be considered for inclusion.
- Descriptive observational study designs including case series, individual case reports and descriptive cross-sectional studies for inclusion
- Qualitative studies that focus on qualitative data include, but are not limited to, designs such as phenomenology, grounded theory, ethnography, qualitative description, action research and feminist research.
- Mixed-method studies
- Systematic reviews that meet the inclusion criteria will also be considered for mapping and contextualising purposes, but their results will not be synthesised.
- Text and opinion papers but only those cited by texts that meet the full inclusion criteria

## Methods

The proposed scoping review will be conducted following the JBI methodology for scoping reviews: searching, selecting, extracting and analysis of evidence and presentation of results [36].

### Search strategy

The search strategy will aim to locate both published and unpublished studies. A limited search of MEDLINE and Google Scholar was undertaken to identify articles on the topic. The text words in the titles and abstracts of relevant articles and the index terms used to describe the articles were used to develop a complete search strategy for Ovid MEDLINE (see Supplementary Material 1). The search strategy, including all identified keywords and index terms, will be adapted for each included database and information source. The reference list of all included sources of evidence will be screened for additional studies.

Due to resource issues, only studies published in English, French, Portuguese and Spanish will be included. Studies published since 2013, but not earlier, will be noted to ensure a full 10 years’ worth of work is covered while ensuring the review is feasible to complete. There is a paucity of relevant literature published prior to this time. Papers published in the last 10 years will integrate findings from earlier research.

The databases to be searched include Ovid MEDLINE, Ovid Embase, EBSCO CINAHL and EBSCO PsycINFO. Sources of unpublished studies/grey literature to be searched include the reference list of all included texts. Every effort will be made to ensure that relevant papers are retrieved, including contacting the study authors.

### Study/source of evidence selection

Following the search, all identified citations will be collated and uploaded into *EndNote 20.5/2020 (Clarivate Analytics, PA, USA)* and duplicates removed. They will then be transferred into *Covidence (Veritas Health Innovation Ltd., Victoria, Australia)*. Following a pilot test, titles and abstracts will then be screened for assessment against the inclusion criteria for the review (see Table 1 below). Potentially relevant sources will be retrieved in full, and their citation details will be imported into *Covidence*. The full text of selected citations will be assessed against the inclusion criteria. Reasons for excluding sources of evidence that do not meet the inclusion criteria at the full-text review stage will be recorded and reported in the scoping review. All screening and full-text assessment will be carried out by two or more independent reviewers. Any disagreements between the reviewers at each stage of the selection process will be resolved through discussion or with an additional independent third reviewer. The search results and the study inclusion process will be reported in full in the final scoping review and presented in a Preferred Reporting Items for Systematic reviews and Meta-Analyses extension for Scoping Reviews (PRISMA-ScR) flow diagram [37].

**Table 1:** Inclusion and exclusion criteria for the review

Inclusion criteria	Exclusion criteria
1. Peer-reviewed journals	1. *Thematically not relevant (only a single condition vs multimorbidity) or no interprofessional collaboration as defined by interprofessional global)*
2. *References available through databases searched*	2. *Research question not suitable (assessment of a complex intervention for multimorbidity, without focus on interprofessional collaboration)*
3. *Freely accessible *via* the University of St. Andrews or NHS Scotland*	3. *Design (comment, letter to the editor, *etc*., not primary research)*
4. *Published in the last 10 years*	4. *Articles which were non-peer-reviewed (such as conference proceedings)*
5. *Available in English, French, Portuguese, or Spanish language*	5. *Articles published earlier than 2013*
6. *Primary focus on multimorbidity and interprofessional collaborative practice*	6. *Language (not English, French, Portuguese or Spanish)*
	7. *Full text not available to NHS Scotland or the University of St. Andrews*
	8. *Animal or lab study*

### Data extraction

Two or more independent reviewers will use a data extraction tool to extract data from papers and grey literature included in the scoping review. The data extracted will include specific details about the participants, concept, context, study methods and critical findings relevant to the review questions.

First, the draft extraction form will be piloted to ensure all the desired information is within the form and to test its feasibility and acceptability. Ten articles will be used in this calibration exercise, including all reviewers involved. Second, the data from those 10 articles will be examined with the intended analysis in mind to ensure the data gathered will fit the planned analysis. Third, if there are no modifications required, following training on how to use the form, to ensure the reliability of the form and the data extractors, all extractors will use the form on the same five full texts to examine the comparability of the data extractors by carrying out a calibration exercise.

A draft extraction form modification list is provided (see Supplementary Material 2). The draft data extraction tool will be modified and revised as necessary while extracting data from each included evidence source. Modifications will be detailed in the scoping review. Any reviewer disagreements will be resolved through discussion or with an additional reviewer/s. If appropriate, authors of papers will be contacted to request missing or other data, where required. All data extracted from every source will be available as a supplementary XLSX file with open access.

The quality of all included texts will be assessed to ensure the quality of any conclusions drawn from the work and to provide scientific rigour to this scoping review. Critical appraisal and quality assurance will be carried out using the most appropriate tool for the method selected: for all qualitative work — the Cochrane risk-of-bias tool for randomised work and the ROBINS-1 for all non-randomised studies. Qualitative papers will be assessed using the Joanna Briggs Institute validated checklist. For mixed-methods studies, the mixed-methods appraisal tool will be used.

### Data analysis and presentation

Simple frequency counts of concepts, populations and characteristics are planned. These will include demographics, diagnostic clusters, professional groups, geographical, policy and clinical contexts.

Descriptive content analysis of qualitative data, including basic data coding, may be undertaken if appropriate. An inductive approach will be taken, as it is anticipated that the evidence may be scarce. This process will involve examining the extracted data and, for each evidence source, listing initial thoughts, possible categories and notes, as they relate to the review subquestions. These will be refined by the review team and will be an iterative process. The categories will be developed into a coding framework.

This coding framework will be applied to all the extracted qualitative data. Two scoping review authors will independently assign all the extracted data to a category. They will meet to discuss any discrepancies. If consensus cannot be achieved, a third reviewer will manage any discrepancies.

Where sufficient homogeneous interventions are applied within a similar context, the pooled odds ratios or standardised mean differences will be calculated and reported using meta-analysis techniques. The assessment of homogeneous interventions will be carried out by two reviewers independently, and should there be discordant views, a third reviewer will make this decision. They will assess the similarity of participants, interventions, comparators, settings, outcomes, study design and risk of bias. The objective of meta-analysis is to estimate a summary average effect. Heterogeneity of the variance in study effect sizes will be assessed using the chi-squared text for heterogeneity. If the *I*^2^ statistic is greater than 55%, then no meta-analysis will be performed. As it is intended to draw conclusions from any such pooling beyond any papers included in the meta-analysis, a random-effects model is planned. Accordingly, the DerSimonian and Laird method of statistical meta-analysis is planned, using RevMan. Sensitivity analysis will explore the impact of excluding studies depending on sample size; methodological quality and variance.

A summary of the included articles will be tabulated, key themes and observations will be charted and themes will be visually displayed [38, 39],

A narrative summary will accompany the results and describe how they relate to the review’s objective and questions.

For quantitative papers, sensitivity analysis will be performed in terms of removing low-quality papers, as identified by the risk-of-bias 2 tool and ROBINS-1, and see if this changes any of the results. This will also be repeated removing randomised papers, and also removing observational studies, to see if the results are sensitive to these changes. Using methods from Langer et al. should there be any thematic synthesis of qualitative results, we will assess whether the synthesis results are sensitive to (1) the exclusion of studies after the application of the critical appraisal tool, (2) the applied qualitative research methodologies and (3) the inclusion of individual studies yielding a larger than an average number of themes [40].

Analysis scripts, notes, charts and figures will be stored on the University of St. Andrews server made available upon reasonable request. Once the scoping review has been published, access to these materials will made open, and the specific URL will be shared.

### Expert consultation

There is broad scholarly consensus that consulting experts within a review is beneficial. This is because it will increase the validity, provide contextual value and provide valuable insights which published literature cannot express [41]. Three main expert groups will be consulted: those with lived experience of caring for or living with multimorbidity, professionals working in health or social care and academics from interprofessional collaboration and multimorbidity.

They will be invited to identify grey literature, advise on any findings’ context and historical aspects and provide personal insights.

## Discussion

This scoping review will provide an overview of the current evidence base of interprofessional collaborative practice used internationally for people living with multimorbidity in health and social care settings. Specifically, it will provide information on existing work examining the specific populations, professional groupings, geopolitical contexts and scientific methods used for IPCP for multimorbidity over the last 10 years. It will allow identification of any gaps regarding knowledge in this area.

A limitation of this review is that it will only gather grey literature from the reference lists of included full-text studies. This may result in publication bias. However, this systematic error will be somewhat corrected by hand searching, checking reference lists of included texts and engaging with topic experts.

This review will be the first part of a study to explore and deepen understanding of IPCP for people living with multimorbidity, examining the barriers and enablers in practice, knowledge, perceptions and experiences in Scottish health and social care.

This research may contribute to health and social care professionals’ daily practice to benefit patient care applicable across all settings. Furthermore, the findings of this research can identify potential areas for standardisation and improvement for leaders and commissioners of the health and social care workforce, as well as policymakers, in preparation for the public health challenge of a population increase in multimorbidity.

## Supplementary Information

Supplementary Material 1. Search strategy.

Supplementary Material 2. Data extraction instrument [42].

Supplementary Material 3. PRISMA-P 2015 Checklist.

## Data Availability

The datasets used and analysed during the current study are available from the corresponding author upon reasonable request.

## Abbreviations

IPCP: Interprofessional collaborative practice
MM: Multimorbidity
NHS: National Health Service

## Glossary

Comorbidity: means that one 'index' long-term condition is the focus of attention, and other health conditions are viewed in relation to this [10].
Multimorbidity: describes someone having two or more long-term (chronic) conditions without any of them holding priority over the others [11].
Interprofessional Collaborative Practice: “when multiple health workers from different professional backgrounds provided comprehensive services by working with patients, their families, carers and the communities to deliver the highest quality of care across settings” [7].
