# Supplementary material for: Interprofessional collaborative practice in health and social care for people living with multimorbidity: a scoping review protocol
Source: Syst Rev. 2025 Jan 2;14:3. doi: 10.1186/s13643-024-02730-x (PMC11697734; doi:10.1186/s13643-024-02730-x)
Supplement: Supplementary file 1 — Supplementary Material 1. Search strategy. [file 13643_2024_2730_MOESM1_ESM.docx]

### Appendix I: Search strategy

**Ovid MEDLINE(R) <1946 to November Week 2 2022>**

**1 exp Multimorbidity/**

**2 (multimorbid$ or multi-morbid$).m_titl,ab.**

**3 (multiple adj2 diseas$).m_titl,ab.**

**4 (multiple adj2 diagnos$).m_titl,ab.**

**5 (multiple adj2 illness$).m_titl,ab.**

**6 (multiple adj2 condition).m_titl,ab.**

**7 (multiple adj2 morbid$).m_titl,ab.**

**8 (coexisting adj2 diseas$).m_titl,ab.**

**9 (coexisting adj2 illness$).m_titl,ab.**

**10 (coexisting adj2 diagnos$).m_titl,ab.**

**11 (coexisting adj2 condition$).m_titl,ab.**

**12 (coexisting adj2 morbid$).m_titl,ab.**

**13 (co-existing adj2 diseas$).m_titl,ab.**

**14 (co-existing adj2 illness$).m_titl,ab.**

**15 (co-existing adj2 diagnos$).m_titl,ab.**

**16 (co-existing adj2 morbid$).m_titl,ab.**

**17 (concurrent adj2 diseas$).m_titl,ab.**

**18 (concurrent adj2 illness$).m_titl,ab.**

**19 (concurrent adj2 diagnos$).m_titl,ab.**

**20 (concurrent adj2 condition$).m_titl,ab.**

**21 (concurrent adj2 morbid$).m_titl,ab.**

**22 (comorbid adj2 diseas$).m_titl,ab.**

**23 (comorbid adj2 illness$).m_titl,ab.**

**24 (comorbid adj2 diagnos$).m_titl,ab.**

**25 (comorbid adj2 condition$).m_titl,ab.**

**26 (comorbid adj2 morbid$).m_titl,ab.**

**27 (co-morbid adj2 diseas$).m_titl,ab.**

**28 (co-morbid adj2 illness$).m_titl,ab.**

**29 (co-morbid adj2 diagnos$).m_titl,ab.**

**30 (co-morbid adj2 condition$).m_titl,ab.**

**31 (co-morbid adj2 morbid$).m_titl,ab.**

**32 multiple comorbid$.m_titl,ab.**

**33 multiple co-morbid$.m_titl,ab.**

**34 case-mix$.m_titl,ab.**

**35 casemix$.m_titl,ab.**

**36 "comorbid*".m_titl,ab.**

**37 co-morbid.m_titl,ab.**

**38 1 or 2 or 3 or 4 or 5 or 6 or 7 or 8 or 9 or 10 or 11 or 12 or 13 or 14 or 15 or 16 or 17 or 18 or 19 or 20 or 21 or 22 or 23 or 24 or 25 or 26 or 27 or 28 or 29 or 30 or 31 or 32 or 33 or 34 or 35 or 36 or 37**

**39 (interprofessional adj2 collaborat$).m_titl.**

**40 exp Interprofessional Relations/**

**41 exp Patient Care Team/**

**42 exp Intersectoral Collaboration/**

**43 exp Cooperative Behavior/**

**44 exp Physician-Nurse Relations/**

**45 exp Interdisciplinary Communication/**

**46 collaborat$.m_titl,ab.**

**47 communication.m_titl,ab.**

**48 cooperat$.m_titl,ab.**

**49 "co-operat*".m_titl,ab.**

**50 coordinat$.m_titl,ab.**

**51 "co-ordinat$".m_titl,ab.**

**52 Crossdisciplinar$.m_titl,ab.**

**53 "cross-disciplinar$".m_titl,ab.**

**54 (integrated adj2 care).m_titl,ab.**

**55 interdisciplin$.m_titl,ab.**

**56 inter-disciplin$.m_titl,ab.**

**57 interprofession$.m_titl,ab.**

**58 inter-profession$.m_titl,ab.**

**59 interagen$.m_titl,ab.**

**60 inter-agen$.m_titl,ab.**

**61 multiagen$.m_titl,ab.**

**62 multi-agen$.m_titl,ab.**

**63 multidisciplinar$.m_titl,ab.**

**64 multi-disciplinar$.m_titl,ab.**

**65 multiprofession$.m_titl,ab.**

**66 multi-profession$.m_titl,ab.**

**67 networking.mp.**

**68 partnership.mp.**

**69 (partnership adj2 work$).m_titl,ab.**

**70 (shared adj2 work$).m_titl,ab.**

**71 (shared adj2 decision$).m_titl,ab.**

**72 (joint adj2 decision$).m_titl,ab.**

**73 team$.m_titl,ab.**

**74 transdisciplinar$.m_titl,ab.**

**75 trans-disciplinar$.m_titl,ab.**

**76 transprofession$.m_titl,ab.**

**77 trans-profession$.m_titl,ab.**

**78 39 or 40 or 41 or 42 or 43 or 44 or 45 or 46 or 47 or 48 or 49 or 50 or 51 or 52 or 53 or 54 or 55 or 56 or 57 or 58 or 59 or 60 or 61 or 62 or 63 or 64 or 65 or 66 or 67 or 68 or 69 or 70 or 71 or 72 or 73 or 74 or 75 or 76 or 77**

**79 (outcome and process assessment).mp. [mp=title, book title, abstract, original title, name of substance word, subject heading word, floating sub-heading word, keyword heading word, organism supplementary concept word, protocol supplementary concept word, rare disease supplementary concept word, unique identifier, synonyms]**

**80 exp "Outcome and Process Assessment, Health Care"/**

**81 effect$.m_titl,ab.**

**82 impact.m_titl,ab.**

**83 change.m_titl,ab.**

**84 outcome$.m_titl,ab.**

**85 80 or 81 or 82 or 83 or 84**

**86 exp Patients/**

**87 ward$.m_titl,ab.**

**88 Hospitals/ not animal.mp. [mp=title, book title, abstract, original title, name of substance word, subject heading word, floating sub-heading word, keyword heading word, organism supplementary concept word, protocol supplementary concept word, rare disease supplementary concept word, unique identifier, synonyms]**

**89 exp Community Health Centers/**

**90 exp Community Health Services/**

**91 exp Community Health Nursing/**

**92 exp Community Mental Health Services/**

**93 exp Community Pharmacy Services/**

**94 exp Home Care Services/**

**95 exp General Practice/**

**96 exp Family Practice/**

**97 exp General Practitioners/**

**98 exp Physicians, Family/**

**99 exp Physicians, Primary Care/**

**100 exp Primary Health Care/**

**101 exp Office Visits/**

**102 exp Primary Care Nursing/**

**103 exp Hemodialysis, Home/ or exp Home Care Agencies/ or exp Home Environment/ or exp Home Health Aides/ or exp Home Health Nursing/ or exp Home Infusion Therapy/ or exp Home Nursing/ or exp "Hospital to Home Transition"/ or exp Parenteral Nutrition, Home/**

**104 exp Social Support/**

**105 86 or 87 or 88 or 89 or 90 or 91 or 92 or 93 or 94 or 95 or 96 or 97 or 98 or 99 or 100 or 101 or 102 or 103 or 104**

**106 38 and 78 and 85 and 105**

**107 106 not (Animals/ not (Animals/ and Humans/))**

**108 limit 107 to (yr="2012 -Current" and (English or French or Portuguese or Spanish))**
