# Supplementary material for: Interprofessional collaborative practice in health and social care for people living with multimorbidity: a scoping review protocol
Source: Syst Rev. 2025 Jan 2;14:3. doi: 10.1186/s13643-024-02730-x (PMC11697734; doi:10.1186/s13643-024-02730-x)
Supplement: Supplementary file 2 — Supplementary Material 2. Data extraction instrument [42]. [file 13643_2024_2730_MOESM2_ESM.docx]

### Appendix II: Data extraction instrument

The JBI extraction instrument from Appendix 11.1 of the *JBI Manual for Evidence Synthesis* will be used and modified.^42^

It will be modified to include the following criteria:

a. Author(s)

b. Year of publication

c. Origin/country of origin (where the study was published or conducted)

d. Aims/purpose

e. Study population, diagnoses, demographics, and sample size (if applicable)

f. Methodology/methods

g. Intervention type, comparator, and details of these (including which professional groups involved)

h. Duration of the intervention (if applicable)

i. Outcomes and details of these (e.g. how measured) (if applicable)

j. Key findings

k. Geographical setting (urban/rural)

l. policy context (any that are relevant)
